# Upper ocean oxygenation, evolution of RuBisCO and the Phanerozoic succession of phytoplankton

**DOI:** 10.1016/j.freeradbiomed.2019.05.006

**Published:** 2019-08-20

**Authors:** Rosalind E.M. Rickaby, M.R. Eason Hubbard

**Affiliations:** Department of Earth Sciences, University of Oxford, South Parks Road, Oxford, OX1 3AN, UK

## Abstract

Evidence is compiled to demonstrate a redox scale within Earth's photosynthesisers that correlates the specificity of their RuBisCO with organismal metabolic tolerance to anoxia, and ecological selection by dissolved O_2_/CO_2_ and nutrients. The Form 1B RuBisCO found in the chlorophyte green algae, has a poor selectivity between the two dissolved substrates, O_2_ and CO_2_, at the active site. This enzyme appears adapted to lower O_2_/CO_2_ ratios, or more “anoxic” conditions and therefore requires additional energetic or nutrient investment in a carbon concentrating mechanism (CCM) to boost the intracellular CO_2_/O_2_ ratio and maintain competitive carboxylation rates under increasingly high O_2_/CO_2_ conditions in the environment. By contrast the coccolithophores and diatoms evolved containing the more selective Rhodophyte Form 1D RuBisCO, better adapted to a higher O_2_/CO_2_ ratio, or more oxic conditions. This Form 1D RuBisCO requires lesser energetic or nutrient investment in a CCM to attain high carboxylation rates under environmentally high O_2_/CO_2_ ratios. Such a physiological relationship may underpin the succession of phytoplankton in the Phanerozoic oceans: the coccolithophores and diatoms took over the oceanic realm from the incumbent cyanobacteria and green algae when the upper ocean became persistently oxygenated, alkaline and more oligotrophic. The facultatively anaerobic green algae, able to tolerate the anoxic conditions of the water column and a periodically inundated soil, were better poised to adapt to the fluctuating anoxia associated with periods of submergence and emergence and transition onto the land. The induction of a CCM may exert a natural limit to the improvement of RuBisCO efficiency over Earth history. Rubisco specificity appears to adapt on the timescale of ∼100 Myrs. So persistent elevation of CO_2_/O_2_ ratios in the intracellular environment around the enzyme, may induce a relaxation in RuBisCO selectivity for CO_2_ relative to O_2_. The most efficient RuBisCO for net carboxylation is likely to be found in CCM-lacking algae that have been exposed to hyperoxic conditions for at least 100 Myrs, such as intertidal brown seaweeds.

## Highlights

• Dissolved O_2_/CO_2_ selects for a redox scale of phytoplankton Rubisco substrate selectivity and anaerobic metabolic ability.
• Increasing O_2_/CO_2_ induced a positive feedback selecting for red-algal derived plastids at the start of the Mesozoic.
• The relative affinity of RuBisCO for O_2_ and CO_2_ tunes to compensate for environmental O_2_/CO_2_ on timescales of 100–1000 yrs.
• Induction of a CCM relaxes enzyme specificity over ∼ 100 Myrs providing a limit to improvement of RuBisCO selectivity.
• Persistently high O_2_/CO_2_ ratios in restricted intertidal zones selects the most efficient RuBisCO in species lacking a CCM.

## Graphical abstract

**Figure:**
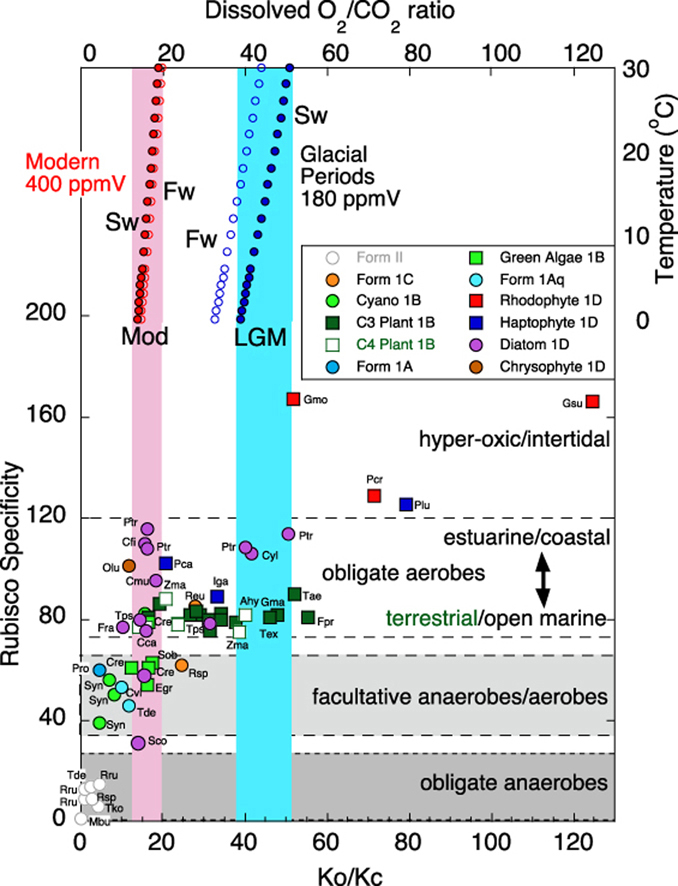

## 1 Aim

This hypothesis paper aims to integrate recent measurements of RuBisCO kinetic parameters across the phytoplankton and terrestrial plants together with data on the physiology, ecology and evolution of oxygenic photosynthesisers. New evidence suggests increased oxygenation of the ocean could have been a selective force in the transformation of the ocean from domination by the green algae to that of the red algal lineage at the Mesozoic. We aim to highlight the contrasting evolutionary trajectories of the green algal lineage with those of the red algal lineage from different ends of the redox spectrum and how selection for different biochemical parameters of the RuBisCO enzyme has worked through time and space. We explore the geological factors that may have triggered a perturbation in the dissolved O_2_/CO_2_ of the ocean leading to an environmental selection towards the mineralising red algal lineage.

## 2 Phanerozoic phytoplankton and upper ocean oxygenation

Three independent lines of evidence demonstrate that the Phanerozoic ocean was dominated by a succession of phytoplankton: microfossils, molecular biomarkers, and molecular clocks for individual clades. The low C28/C29 ratios of the sterane profiles of Paleozoic rocks are most likely driven by early diverging prasinophyte green algae, and chlorophyte green algae that produce high abundances of C29 relative to C27 and C28 sterols as found from a large, phylogenetically based survey of sterol profiles from the kingdom Plantae [1]. The Devonian saw an expansion of more derived prasinophyte algae (Chlorophyta) at the expense of the incumbent phytoplankton as evidenced by an extremely high sterane/hopane ratio in sedimentary lipids [2], and elevated C28:C29 sterane ratios [1,3]. The later and more derived groups of green algae produce a greater abundance of C28 relative to C27 and C29 sterols [1]. Later, the Mesozoic Ocean was taken over by the chlorophyll *a+c* containing phytoplankton of the haptophytes (e.g. coccolithophores) and heterokont (e.g. diatom) lineages, whose plastids are derived from red algae (Rhodophyta) via secondary endosymbiosis [[4], [5], [6]]. In each case the larger cell sizes of the phytoplankton, and in the latter, the addition of mineralising skeletons, added power to the biological pump of carbon and nutrients from the surface ocean to the deep, propagating oxygenation and with ramifications throughout the ecosystem.

**Fig. 1:**
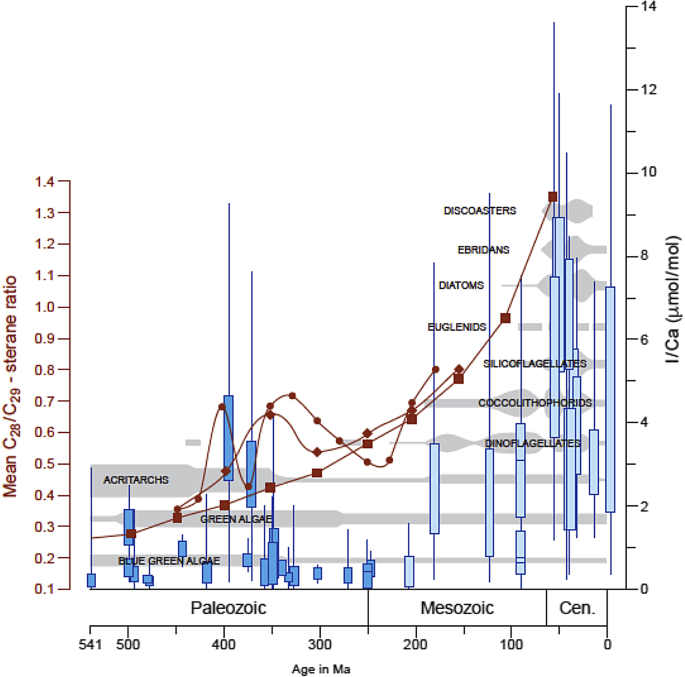
A comparison of algal biomarker records from rock and oil samples with the I/Ca record of upper ocean oxygenation. The C28/C29-sterane ratio of 500 rock samples are plotted, averaged in 50 Myr steps (rhomboids), and in 25 Myr steps (filled circles) compared with 400 oil samples (squares) analysed by Grantham and Wakefield [8] and presented in Schwark and Empt, [2]. Candlestick plot showing ranges of I/Ca values for Paleozoic (dark blue) and Meso-Cenozoic (light blue) from Lu et al., [7]. Boxes mark the 25th and 75th percentiles of values at each locality, and the whiskers show the maximum and minimum. Also shown in grey bars are the inferred abundances of different algal groups throughout the Phanerozoic. (For interpretation of the references to colour in this figure legend, the reader is referred to the Web version of this article.).

Recent evidence based on I/Ca in carbonates, a redox proxy sensitive to suboxia, identified an excursion in recorded I during the Devonian and a step change at 200 Ma, coincident with each of these micro-faunal revolutions [7]. Elevated I/Ca indicates increased ocean oxygenation, and is interpreted as a deepening of the oceanic oxygen minimum zone at each of these times achieving more persistently oxygenated modern day conditions at the Paleozoic-Mesozoic transition. This new record of I/Ca correlates with the abundance of the biomarker C28/C29 steranes, markers of the radiation in more derived green algae (Chlorophyta and Streptophyta) and the succession of the modern phytoplankton groups from a compilation of rock and oil samples (Fig. 1 [2,5,6]). Such a similarity between these two very different datasets founded on contrasting samples and geochemical analyses is strongly suggestive that there could be a common driver to both, such as an increase in surface water oxygenation.

## 3 Chlorophyll *a+c* algae have a higher RuBisCO specificity than chlorophyll *b* algae

The form and specificity of Ribulose‐1,5‐bisphosphate carboxylase/oxygenase (RuBisCO, EC 4.1.1.39), the enzyme that catalyzes CO_2_ fixation during oxygenic photosynthesis, is also transformed across the open ocean upon the transition from a chlorophyll *b* to a chlorophyll *a+c* algal lineage dominated assemblage.

It is thought that all Forms of RuBisCO arose from a Form III RuBisCO within an Archaean methanogen [[9], [10], [11]]. This ancestral form gave rise to all other forms through a complex history involving a number of horizontal and vertical gene transfers [9]. Evolution within Proteobacteria diversified Form I into IA, IC and ID, and within cyanobacteria Form I evolved into Form IB. Most algae and all higher plants contain Form I RuBisCO, a 560 kDa holoenzyme of eight 50–55 kDa large (LSU) and eight 12–18 kDa small (SSU) subunits [12]. Less prevalent is Form II RuBisCO, a dimer of two LSU found in peridinin-containing dinoflagellates and some prokaryotes [13]. Only Form I and II RuBisCO are used for oxygenic photosynthesis and Form I is responsible for the bulk of this carbon fixation.

Form I RuBisCO can be further divided into subforms IA, IB, IC and ID. Form IA is found in α-cyanobacteria such as *Prochlorococcus* spp., while Form IB is found in higher plants, green algae and β-cyanobacteria. Form IC is found in some photosynthetic bacteria e.g. *Rhodobacter sphaeroidea* and Form ID is found in all non-green eukaryotic algae (i.e. red and chromist algae, except Form II-containing dinoflagellates) [14].

All Form I enzymes are structurally similar with 422 symmetry (tetragonal-trapezoidal crystal structure) and a core consisting of four LSU dimers (L_2_) arranged around a four-fold axis, capped at each end with four SSUs [13]. Forms IA – ID can be differentiated according to their amino acid sequence. Forms IA and IB are about 80% similar, as are the forms IC and ID. Between Forms IA/B and IC/D there is only about 60% sequence similarity [15,16]. Despite the different forms of I Rubisco, they all have the same functional active site [17].

During oxygenic photosynthesis, RuBisCO catalyzes two competitive reactions; fixation of CO_2_ for photosynthesis (carboxylation) and energy wasting photorespiration using O_2_ (oxygenation). The ability of a particular RuBisCO to discriminate between the non‐polar, structurally similar substrates CO_2_ and O_2_ is determined by the kinetic properties of the enzyme, denoted as the specificity factor (Ω):

(1) Ω = (V_c_K_o_/V_o_K_c_)

where V_c_ and V_o_ are maximal velocities of the carboxylase and oxygenase reactions and K_c_ and K_o_ are the Michaelis constants for the susbtrates CO_2_ and O_2_. The carboxylation:oxygenation efficiency of the net reaction must also account for the CO_2_ and O_2_ concentrations at the catalytic site of the enzyme:

(2) carboxylation/oxygenation = (V_c_K_o_/V_o_K_c_)*([CO_2_]/[O_2_])

**Fig. 2:**
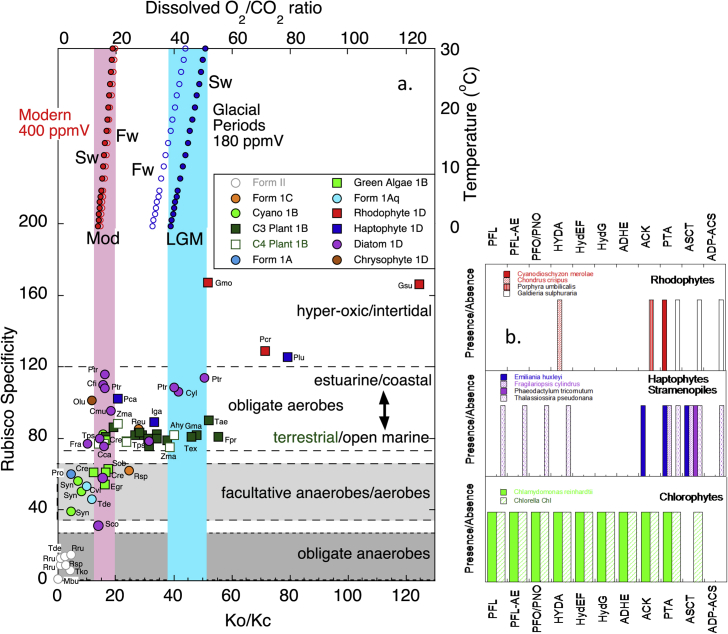
a) The sensitivity of the equilibrium dissolved ratio of O_2_/CO_2_ (CO_2_ [22]) (O_2_ [23]) concentrations to temperature and salinity (S; 0, open circles, and 35 ppt, closed circles) for the modern (with an atmosphere of 400 ppmV) compared to that at the LGM with invariant O_2_ but a CO_2_ atmosphere of 180 ppmV. This environmental O_2_/CO_2_ provides a calibration for the redox gradient to RuBisCO of different algal groups and their ecology showing the relative substrate affinities K_o_/K_c_ of RuBisCO as a first order determinant of RuBisCO specificity. Species abbreviation labels Rhodophyta: Gsu *Galdieria sulfiraria*, Gmo *Griffithsia monilis*, Pcr *Porphyridium cruentum*, Haptophytes: Plu *Pavlovale lutheri*, Pca *Pleurochrysis carterae*, Iga Is*ochrysis galbana*, Heterokonts: *Phaeodactylum tricornutum*, Cyl *cylindrotheca* spp, Cfu *Cylindrus fusiformis*, Cmu *Chaetoceros muellerae*, Chrysophyte: Olu *Olisthodiscus luterae*, Fra *Fragilariopsis* sp, Cca *Chaetoceros calcitrans*, Tps *Thalassiosira pseudonana*, Sco: *Skeletonema costatum*, Green algae: Cre *Chlamydomonas reinhardtii*, Sob *Scenedesmus obliquus*, Egr *Euglena gracilis*, Cyanobacteria: Syn *synechococcus*, Pro *Prochloroccus* Plants: C3: *Triticum aestivium* C4 *Zea Mays,* Anaerobes: Tde *Thiobacillus denitrans*, Rsp *Rhodobacter sphaeroides*, Rru *Rhodospirillum rubrum*, Mbe *Methanococcoides burtonii*, Tko *Thermococcus kodakarensis*. Also labeled are mean ecologies of different groups of algae ranging from obligate anaerobes, through facultative anaerobes to obligate aerobes and hyperoxic tolerant. b) The number of anaerobic metabolic pathways in the genomes (PFL, PFL-AE, PFO/PNO, HYDA, HydEF, HydG, ADHE, ACK, PTA, ASCT, ADP-ACS) of the labeled organisms where RuBisCO specificity has also been determined taken from Atteia et al., [24]. (For interpretation of the references to colour in this figure legend, the reader is referred to the Web version of this article.).

RuBisCO kinetic characterization from a diversity of organisms shows specificities that range from about 4 to 240 [[18], [19], [20], [21]]. As seen in Fig. 2, a replacement of Form IB-containing green algae and β-cyanobacteria across the ocean, by Form ID-containing haptophytes and heterokonts represents an approximate doubling in RuBisCO specificity [ [[18], [19], [20]], Supplementary Table 1] in the open ocean.

## 4 Organisms with higher specificity RuBisCOs are selected by higher environmental O_2_/CO_2_

All else being equal, according to equation (2), an increase in the O_2_/CO_2_ ratio in the environment will decrease carboxylation relative to oxygenation for a given RuBisCO and net photosynthetic efficiency. Phytoplankton may adapt to such an environmental pressure by development of a carbon concentrating mechanism (CCM) to internally elevate CO_2_ relative to O_2_ around RuBisCO and restore net carbon fixation rates [[21], [22], [23]], by expression of a higher specificity RuBisCO should two different enzymes be available in the genome, or by improvement of their RuBisCO selectivity.

Many extant aquatic photosynthesisers possess a CCM [21,25,26]. Key constituents of the CCM include: (i) plasma- and chloroplast-membrane inorganic carbon transporters; (ii) a suite of carbonic anhydrase enzymes in strategic locations; and usually (iii) a microcompartment in the chloroplast in which most Rubisco aggregates (the pyrenoid) [20,27]. Generally, RuBisCO enzymes from algae have evolved a lower affinity for CO_2_ when the algae have adopted a strategy that employs a CCM to help optimise for CO_2_ fixation [20]. The phylogenetic progression in green RuBisCO kinetic properties suggest that RuBisCO substrate affinity for CO_2_ demonstrates a systematic relaxation in response to the origins and effectiveness of a CCM [27]. In land plants, it has been established that positive selection in rbcL emerges coincident with the development of a C_4_ CCM which elevates CO_2_ to almost saturation at the site of RuBisCO [28,29]. This relaxes pressure for RuBisCO to have a high affinity for CO_2_ so K_c_ of C_4_ plants is generally higher than the K_c_ of C_3_ plants. The development of a CCM masks any selective pressure exerted by rising external O_2_/CO_2_ on RuBisCO because it shields the enzyme within a high CO_2_ microenvironment. The extent of relaxation of selective pressure due to the presence of a CCM depends on its efficiency as there is great diversity in the structure and function of CCMs across the aquatic photosynthesisers.

Despite the prevalence of a CCM in aquatic photosynthesisers, there is considerable evidence that environmental O_2_/CO_2_ has frequently exerted a selective pressure on Rubisco specificity, or the use of a higher specificity Rubisco where two forms exist. A higher O_2_/CO_2_ ratio can induce the expression of a higher specificity RuBisCO in a single organism which harbours genes for two forms of RuBisCO of contrasting specificities (e.g. the more efficient Form 1C compared to the lower specificity Form II) such as within the non-sulphur purple photoautotrophic bacteria *Rhodospirillum rubrum* [30] and within the chemolithoautotrophic bacteria *Thiobacillus denitrificans* [31]. Generally, the use of the lower specificity Form II is selected under conditions of high CO_2_ (>1.5%) and low O_2_, in contrast to the induction of the higher specificity Form 1C during photoautotrophic growth at medium levels of CO_2_ (<1.5% CO_2_) and under more aerobic conditions [32].

The compilation of RuBisCO kinetics from extant photosynthesisers suggests that the O_2_/CO_2_ of the environment has also selected for organisms that express a higher specificity RuBisCO. There is a general correlation of RuBisCO specificity with K_o_/K_c_, the ratio of the relative affinities of RuBisCO for the O_2_ and CO_2_ substrates such that a high K_o_ (low affinity for O_2_) relative to a low K_c_ (high affinity for CO_2_) characterizes a highly specific RuBisCO and vice versa. This suggests that changes in relative carboxylation/oxygenation speeds (V_o_/V_c_) of the enzyme are small compared to changes in the relative affinities for the substrates. The K_o_/K_c_ of the RuBisCO in modern marine phytoplankton appears to have adapted to compensate for the dissolved O_2_/CO_2_ ratio in the recent environment [33]. There is a close match between the K_o_/K_c_ = ∼16 for most Form 1D-containing open ocean algae with red-algal derived plastids (e.g. haptophytes, heterokonts) and the modern dissolved O_2_/CO_2_ ratio in sea water (O_2_/CO_2_ ∼ 13 to 19 between 0 and 30 °C). In other words, RuBisCOs of these species display a 16-fold lower affinity for O_2_ than CO_2_ and thus appear to compensate for the 16-fold excess of dissolved O_2_ relative to CO_2_ (Fig. 2). It is not clear why there should be such an apparent tuning of the relative affinities of Rubisco to compensate for the modern environmental ratio of O_2_/CO_2_. One plausible mechanism may be the action of a CCM which acts to elevate the intracellular CO_2_/O_2_ and over time the relative substrate affinities of RuBisCO evolve in response to this compensating intracellular ratio.

This putative selective pressure exerted by environmental O_2_/CO_2_ on the RuBisCO K_o_/K_c_ ratio may, paradoxically, also explain why some photosynthesisers have been found to have a K_o_/K_c_ so extreme as to be apparently tuned to conditions outside the geologically recent range in atmospheric O_2_/CO_2_. During the ice ages of the Pleistocene, atmospheric CO_2_ concentrations have varied in parallel with the temperature fluctuations by ∼100 ppmV [34] but atmospheric O_2_ has stayed near constant [35]. The dissolved O_2_/CO_2_ has fluctuated from ∼13 to 19 in interglacials to ∼39 to 50 during glacial periods (Fig. 2 [33]). Compared to this range, the K_o_/K_c_ of the 1D RuBisCO of two red algae (*Porphyridium cruentum* and *Griffithsia monilis*) and one coastal haptophyte (*Pavlova lutheri*) imply exposure to conditions of hyper-oxia compared to the “norm-oxia” of the modern ocean. Indeed, these species are known to inhabit brackish/intertidal zones which are susceptible to highly elevated O_2_/CO_2_ ratios during daylight hours when the rate of photosynthesis is high and water exchange may be limited [36]. Similarly the thermoacidophiles *Cyanidium caldarium*, *Cyanidium partita* and *Galdiera sulfuraria* likely experience high O_2_/CO_2_ ratios due to both temperature and pH effects on the relative solubilities of these gases in the hot acidic springs of their habitat. Furthermore, amongst the diatoms there is a huge range in specificities likely due to differences in their CCM [19] and their ecology. Some diatoms (*Cylindrotheca fusiformis*, and *Phaeodactylum tricornutum*) have higher specificities (∼110–120) than others. These species have a distinct ecology compared to diatoms reported with lower specificities (∼80), being found in coastal regions, estuaries, mud flats and rock pools compared to the lower specificity open ocean marine diatoms. These highly specific diatom RuBisCOs have a very high K_o_ relative to their K_c_ which may be a result of living in these more restricted hyperoxic coastal/intertidal zones, or even subaerially exposed, compared to open ocean conditions. Similarly two diatom strains of *Skeletonema costatum*, and *Chaetoceros calcitrans* have outstandingly low specificities (specificity of 30.3 and 56.7 respectively). These species are known to have resting stages that persist in oxygen-deficient sediments [37] for decades.

At the other extreme of the environmental scale, the form II RuBisCO of *Methanococcoides burtonii*, *Thermococcus kodakarensis*, *Hydrogenovibrio marinus*, *T. denitrificans*, *Rhodobacter sphaeroides* and *Rhodospirillum rubrum* is expressed only under anaerobic conditions i.e. with extremely low O_2_/CO_2_ ratios. Within this context, the lower K_o_/K_c_ (4–8) of the cyanobacteria RuBisCO compared to the RuBisCO of green algae, haptophytes and heterokonts is consistent with the ability of the β-cyanobacteria to flourish under eutrophication. Such an observation may be supported by the correlation of abundance of microbial carbonates in the geological record with inferred periods of a more poorly oxygenated ocean [38].

This analysis reveals that organisms containing the 1D Form RuBisCO within the chlorophyll *a+c* algae are better adapted to an open ocean environment with an O_2_/CO_2_ ratio (∼16 to 35) that is approximately double that to which the RuBisCO of the cyanobacteria appears to be adapted (O_2_/CO_2_ ratio of 4– to 8). This further supports the hypothesis that a step change in upper ocean oxygenation contributed to the changing success of these different algal groups across the Paleozoic/Mesozoic boundary. Should the cyanobacteria K_o_/K_c_ be tuned to the Paleozoic dissolved O_2_/CO_2_ ratio in the oceans, before the haptophytes and heterokonts took over then atmospheric compositions at that time, could have fluctuated around 10 to 20% O_2_ and 400 to 800 ppm CO_2_ when cyanobacteria were dominant. Even if this link between the K_o_/K_c_ and the O_2_/CO_2_ of the environment is only qualitative, the K_o_/K_c_ does provide some indication of ecological O_2_/CO_2_ ratios and is a first order determinant of RuBisCO specificity.

## 5 Higher specificity RuBisCOs are selected by organisms with aerobic physiology

In addition to environmental O_2_/CO_2_ ratios exerting a selective influence on organisms harbouring different specificity RuBisCOs, there is also a correlation between the physiological adaptation of the photosynthetic organism to aerobic/anaerobic conditions and RuBisCO specificity (Fig. 2). Obligate anaerobes, or facultative anaerobes which express a form II RuBisCO under anaerobic conditions, all have the lowest RuBisCO specificities (between 1 and 16). There is then a distinction between a group containing the higher specificity 1B land plants and 1D containing haptophytes and heterokonts (obligate aerobes) with a specificity between 80 and 120, compared to the group containing the 1B containing green algae and cyanobacteria (facultative anaerobes) with a specificity between 30 and 60. When investigating the differences between these photosynthesizing organisms, a distinctive physiology for the green algae and cyanobacteria emerges compared to other oxygenic autotrophs. They all have the ability to undergo indirect water photolysis to generate H_2_ if grown under anaerobic or low sulfate conditions. Anaerobic metabolic pathways allow unicellular organisms to tolerate or colonize anoxic environments. Green algae, such as *Chlamydomonas reinhardtii*, and *Scenedesmus obliquus*, all have the ability to ferment their plastidic starch to a variety of end products including acetate, ethanol, formate, glycerol, lactate, H_2_ and CO_2_. Cyanobacteria, depending on the species, utilize both nitrogenases and hydrogenases in the pathway of H_2_ production [39], whereas the green algae rely solely on hydrogenases [40]. Nitrogenases have the advantage that they act unidirectionally, whereas hydrogenases are bidirectional [41]. The high O_2_ sensitivity of both enzymes requires the separation of H_2_ evolution and CO_2_ fixation, temporally or spatially.

An overview of the presence of anaerobic metabolic pathways from whole genome analysis confirms such a gradient to the O_2_ tolerance of these physiologies ([24]; Fig. 2b). The red algae may be considered an “oxic” endmember to the algae. They are nearly devoid of any of the pathways involved in anaerobic metabolism. They also appear to have undergone a significant genome reduction in their evolutionary history, which could be responsible for the loss of the ancestral anaerobic pathways from the primary endosymbiosis [42]. Similarly the haptophytes and heterokonts are also lacking many of the anaerobic metabolic genes found in the green algae. In a parallel to the large diversity of diatom RuBisCO specificity, the greatest diversity in anaerobic gene presence is also found among the diatoms. By contrast, many green algae have an abundance of anaerobic pathways. Some were lost via gene reductions (such as in *Ostreococcus tauri*), but *C. reinhardtii* is adapted to both aerobic and anaerobic conditions. In a survey on different algal groups, redox-regulation of some parts of the Calvin Benson Cycle was also found to be variable with the greatest degree of regulation in green algae, but there was little or no redox-regulation in a red alga or in most lineages with red-algal derived plastids (including the diatoms) [43].

It was from within the Chlorophyte green algae, through the sister group of the Charophyte green algae, that the land plants emerged with their facultative anaerobic metabolism capable of living on land and becoming truly complex multicellular organisms (defined by three-dimensional body plans and multiple cell types). Meanwhile the red algal lineages have been limited in stature, multicellularity and ability to make roots [44] and were restricted to marine environments. The first steps onto land would have required the ability to differentiate cells to make roots to obtain nutrients from sediments or soils in periodically inundated and anoxic soils (e.g. Refs. [45,46]). Indeed it may have been the cellular differentiation into roots/shoots versus leaves harbouring RuBisCO that allowed the physical segregation between the anaerobic metabolic pathways in the roots and the chloroplasts containing RuBisCO in the leaves that allowed the plant RuBisCOs to make the step change towards a higher RuBisCO specificity, indicative of an aerobic environment, in the leaves.

## 6 Direction of evolution of RuBisCO specificity and nutrient requirement for a CCM

Eukaryotes with a green plastid possess Form IB Rubisco thought to have arisen through endosymbiosis of a Form IB containing cyanobacteria. Within this lineage therefore, the Form 1B RuBisCO improved its specificity in response to increasing O_2_/CO_2_ ratios over time, as seen in the land plants compared to the green algae and cyanobacteria. The emergence of a CCM was required to generate high intracellular CO_2_/O_2_ to maintain photosynthetic efficiency with the poorer specificity RuBisCO found in the cyanobacteria. These CCMs arose relatively late in geological time, ∼420 Ma, after CO_2_ concentrations in the atmosphere and ocean declined from their initially high levels and dissolved O_2_ levels rose [33,47].

It is hard to decipher the direction of evolution of the kinetics of RuBisCO within the form 1D RuBisCO containing lineages. Eukaryotes with a red plastid have Form ID that was originally derived from a γ-proteobacteria [9]. The red algae putatively diverged from the eukaryote tree of life ∼1.1 billion years ago and provided the plastids in the secondary endosymbiotic event that gave rise to the heterokonts and haptophytes. Did the haptophytes and heterokonts inherit a relatively low-specificity RuBisCO from the ancestral rhodophyte via secondary endosymbiosis, which has been retained in most extant haptophyte/heterokont species? Was a lower specificity RuBisCO of the haptophytes and heterokonts then shielded from the evolving environment by emergence of CCM pathways in response to rising O_2_/CO_2_? Did this lower specificity RuBisCO then become more specific in other species, such as *Pavlova lutheri,* that lack a CCM and inhabit higher O_2_/CO_2_ environments? An increase in RuBisCO specificity in the Pavlovales may have occurred concurrently with the selection of a higher RuBisCO specificity in red algae (as Fig. 2 shows *P. lutheri* has closest K_o_/K_c_ to the red algal species). Alternatively, did the haptophytes and stramenopiles inherit an already highly specific RuBisCO from the rhodophytes, which then relaxed under the persistent induction of a CCM elevating internal CO_2_/O_2_ in the haptophytes and heterokonts (as proposed by Young et al. [48])?

Regarding the direction of evolution of RuBisCO specificity in the different lineages, three lines of evidence suggest that the latter hypothesis may be the more likely scenario i.e. that the haptophytes and stramenopiles inherited an already highly specific RuBisCO which then relaxed in specificity over time. Firstly, the ancient fossil record of the red algae, Bangiomorpha, places them as continuous inhabitants of the hyperoxic peri-to supra-tidal environment [49]. The peri-tidal zone is distinct for harbouring hyperoxic conditions of elevated O_2_ and much diminished CO_2_ concentrations during daily light-driven photosynthesis [36], and the supratidal zone sees highly elevated atmospheric O_2_/CO_2_ ratio with million fold faster diffusion rates. Another representative of the red algae, the Porphyra and its ancestors, have competed successfully in this dynamic and severe intertidal environment for over a billion years [44]. Similarly, the relatively morphologically simple Pavlovales have always been restricted to near-shore, brackish, or freshwater environments often with semibenthic modes of life, and this may mirror the ancestral ecological strategy of the Paleozoic haptophytes [5]. Even under a poorly oxygenated atmosphere, it is likely that these restricted coastal zones where photosynthesis was rife were consistently hyperoxic. Over this billion year timeframe, these hyperoxic conditions exerted a selection pressure beyond that of typical ocean conditions and selected for RuBisCOs that were better adapted to these rather more extremely oxygenated conditions, a trait inherited by the secondary endosymbiotic lineages. To obtain a competitive edge in this environment, algae could have additionally induced a CCM. It is through the persistent induction of a CCM to elevate internal CO_2_/O_2_ ratios that eventually the RuBisCO specificity relaxed during the speciation events that founded the lineages including the haptophytes and heterokonts.

**Fig. 3:**
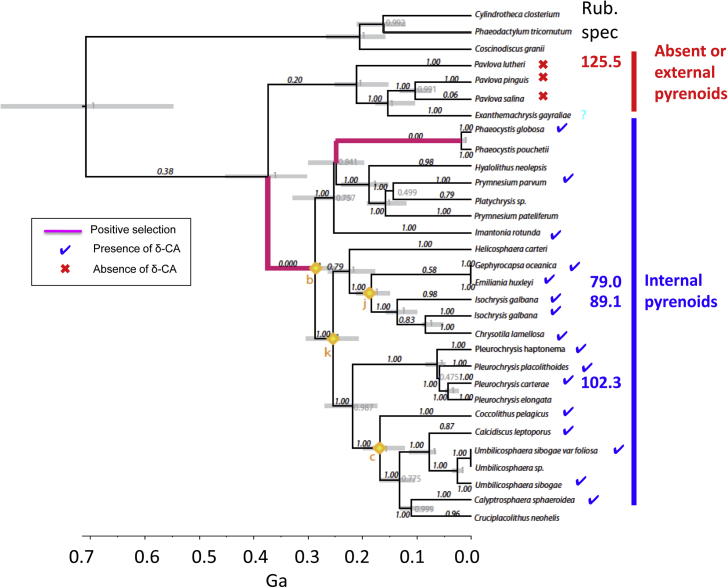
Phylogenetic Tree for RbcL within Haptophyta showing branches under positive selection (magenta) and those with no positive selection (black) adapted from Young et al., [48]. Black numbers above branch is statistical significance (p-value) of positive selection after a likelihood ratio test comparing nested models and using Bonferroni correction. Grey bars denote 95% confidence intervals for date estimates and grey numbers are posterior probability values. Yellow diamonds with corresponding letter are fossil calibration dates. Also indicated is the presence/absence of some components of a CCM with a blue tick for the presence of a δCA (methodology from Heureux et al. [20]), and a red bar for the absence or presence (blue) of a pyrenoid (Eason Hubbard unpublished; *P. lutheri (*now *Diacronema lutheri)* [50,51] *Pavlova pinguis* [52,53], *Pavlova salina* (now *Rebecca salina*) [51,53], *Exanthemachrysis gayraliae* [51,54], *Phaeocystis globosa* [55], *Phaeocystis pouchetii* [56], *Hyalolithus neolepis* (now *Prymnesium neolepis*) [57], *Prymnesium parvum* [58], *Prymnesium patelliferum* (now *Prymnesium parvum* haploid stage) [59], *Imantonia rotunda* (now *Dicrateria rotunda*) [60], *Gephyrocapsa oceanica* [61], *Helicosphaera carteri* (Eason Hubbard unpubl), *Emiliania huxleyi* [62,63], *Isochrysis galbana* [64], *Chrysotila lamellosa* [65], *Pleurochrysis placolithoides* [66], *Pleurochrysis carterae* [67], *Pleurochrysis elongate* [68], *Coccolithus pelagicus* [69], *Calcidiscus leptoporus* (Eason Hubbard unpubl), *Umbilicosphaera sibogae* var *foliosa* [70], *Calyptrosphaera sphaeroidea* (now *Holococcolithophora sphaeroidea*) [71], *Cruciplacolithus neohelis* [72]. Also indicated is the RuBisCO specificity in numerical values where data is available. (For interpretation of the references to colour in this figure legend, the reader is referred to the Web version of this article.)

The strongest signal of positive selection in haptophyte RuBisCOs is at the divergence between the Pavlovophycaeae and Prymnesiophycaeae [48] with a step change in RuBisCO specificity (from 125 to 90) between respective representatives *P. lutheri* and *I. galbana* (Fig. 3). Distinct differences in RuBisCO specificities within the haptophyte lineage RuBisCO correlate with the formation of a pyrenoid and/or presence of a CCM [20]. *Pavlova lutheri* has a low cellular affinity for carbon, negligible change in this affinity when adapted to high or low external carbon conditions (Rae et al., unpubl data) and lacks a pyrenoid [50,51], suggesting that it has no CCM [20]. By contrast both *Isochrysis galbana* and *Pleurochrysis carterae* contain a pyrenoid [50,64,67] and are known to possess CCMs (including carbonic anhydrases) and lower specificity RuBisCOs compared to *P. lutheri*.

**Fig. 4:**
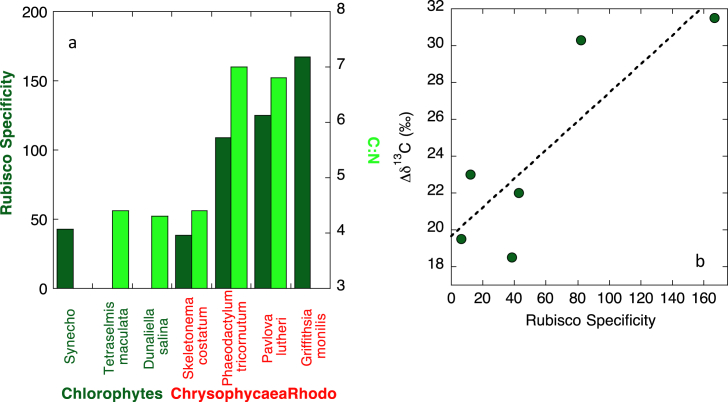
a) Comparison between RuBisCO specificity (dark green bars) and the Redfield ratio (C:N measured in cells under exponential growth, light green bars [73]). These are data measured on a wide variety of species under the exact same conditions, important for internal consistency given the propensity for plasticity in the Redfield ratio. Here the Redfield ratio is interpreted to reflect nutrient efficiency of C fixation and C:N is higher when carbon fixation is more nutrient efficient and requires less proteins of a CCM. B) The relationship between higher RuBisCO specificity and larger carbon isotopic fractionation of the RuBisCO enzyme *in vitro* taken from Tcherkez et al. [74], and updated with data for *S. costatum* from Boller et al., [75]. (For interpretation of the references to colour in this figure legend, the reader is referred to the Web version of this article.).

The winners of the competition for success in the open ocean then derives from the nutrient efficiency with which algal lineages can maintain high rates of carboxylation relative to oxygenation. Across a rise in ocean O_2_/CO_2_ at the Mesozoic, as a result of their poorer RuBisCO specificity, β-cyanobacteria need to invest greater nutrient resource in fixing carbon via a more efficient CCM than the haptophytes and heterokonts with a more highly specific RuBisCO and lesser need of investment in proteins for a CCM to succeed within this niche. A hint of this higher nutrient requirement for C fixation in species with a lower RuBisCO specificity is afforded by a comparison of the Redfield ratio of a range of species measured under identical laboratory conditions in the same study (Fig. 4a). There is such plasticity to the Redfield ratio that direct comparison across a broad range of species from the exact same conditions is the only way to obtain a direct comparison. If a higher C:N reflects a higher efficiency C fixation process per protein expressed, then species with higher RuBisCO specificities indeed obtain greater C fixation rates per nitrogen fixed as a result of requiring less proteins for the CCMs. In addition, green seaweeds from the upper intertidal zone but same location have been found to have lower C:N ratios (∼10) than both red (∼13) and brown seaweeds (∼16) for the lower intertidal zone [76] suggestive that these different Redfield ratios may be more broadly characteristic of these algal lineages. Upon oxygenation and increasing oligotrophy of the open ocean, haptophytes and heterokonts could outcompete the green algae and β-cyanobacteria in terms of net carboxylation relative to oxygenation per nutrient required.

Such nutrient selection and open ocean or coastal selection of CCM efficiency is evident amongst the different groups of cyanobacteria, compensating for a lesser RuBisCO efficiency. The open ocean α-cyanobacteria with form IA RuBisCOs have a restricted suite of HCO_3_^−^ accumulation processes and little capacity to acclimatize to decreased inorganic C availability [47,77,78] The oceanic α -cyanobacteria may have developed a physiology where they may not have the ability to acquire or induce high-affinity carbon transport systems, and in some species no active CO_2_ uptake system may be present which makes them nutrient efficient and well adapted to the open ocean. On the other hand, many β-cyanobacteria have the ability to induce various CO_2_ and HCO_3_^−^ transport systems as their environmental conditions change [47,77,78] and so flourish in coastal nutrient rich zones. Indeed the sensing of oxygen may play a key role in the induction of a CCM in the cyanobacteria [79].

It is worth noting that this consideration above yields contrasting nutrient gradients for CCMs to succeed in different O_2_/CO_2_ environments between the chlorophyte and chromalveolate lineages. Amongst the green algae with a poorly specific RuBisCO, a great energetic or nutrient investment in a CCM is necessary to inhabit conditions of elevated O_2_/CO_2_. By contrast, amongst the haptophytes and heterokonts, the greater nutrient requirement for a CCM, which over time relaxes RuBisCO specificity, allows them to extend their ecology into lower O_2_/CO_2_ environments.

A second argument to support the red algae having consistently expressed high specificity RuBisCOs rather than evolving towards a higher specificity RuBisCO under rising O_2_/CO_2_ comes from C isotopic fractionation. A more highly specific RuBisCO binds the CO_2_ substrate more tightly and thus results in a larger kinetic fractionation associated with C fixation compared to a lower specificity RuBisCO ([74], Fig. 4b). The larger C isotopic swings of oceanic δ^13^C in the Neoproterozoic which dampen towards the modern day [80] could either reflect variations in the burial rate of isotopically light carbon (e.g. Ref. [81]) or burial of different sources of organic carbon with more extreme C isotopic signatures than those of the modern day. If burial of organic matter oscillated between the much lighter isotopic carbon produced by the highly specific RuBisCO in the coastal Pavlovales and or Porphyra, and the much isotopically heavier organic matter of the cyanobacteria [82], this could contribute to the higher amplitude oscillations of the early part of the δ^13^C record (see Figure 2c of [81]). The oceanic δ^13^C oscillations became damped as CCMs were induced and the more intermediate specificity RuBisCOs of the terrestrial plants, haptophytes and heterokonts became increasingly prevalent throughout the Phanerozoic contributing to burial of organic matter with less extreme carbon isotopic compositions due to the smaller isotopic fractionations factors of these more intermediate specificity RuBisCOs. There are hints that some seaweed δ^13^C and *P. lutheri* δ^13^C may be extremely isotopically light [83,84] which could be consistent with a large carbon isotope discrimination factor of a highly specific RuBisCO.

Thirdly, there is little difference in RuBisCO specificity between the cyanobacteria (the primary endosymbiont) and the chlorophyte green algae nor between the red alga, *P. cruentum* (a putative secondary endosymbiont) and the haptophyte *P. lutheri* suggestive that the ancestor of the endosymbiont and secondary lineages bearing that endosymbiont are similar in their RuBisCO specificity. Furthermore, the positive selection and change in RuBisCO specificity are coincident at the speciation events [48]. This might suggest that the process of endosymbiosis itself did not change the specificity, and that it was the later environmental change around the RuBisCO that effected a change in the RuBisCO of the land plants and that of the haptophytes and heterokonts.

## 7 Tempo of evolution of RuBisCO kinetics

### 7.1 Slow to generate specificity

Despite the general correlation of RuBisCO specificity with relative substrate affinity across the continuum of change in K_o_/K_c_, there are discrete groups of RuBisCO specificities characterized by a range of K_o_/K_c_ values (Fig. 3). This is suggestive that K_o_/K_c_ may evolve very quickly, and in response to environmental or physiological conditions, but a change in specificity takes much longer. If net carboxylation is the single rate-limiting step for the growth and replication of a single-celled photosynthesizing microorganism, then strong positive selection will be exerted upon the enzyme that catalyzes this step i.e. RuBisCO. A strong selection pressure from an increasing O_2_/CO_2_ at the active site can impart drastic improvements in a short period of time, yielding an evolved enzyme that is no longer the weak link in the metabolic network of the cell, hence step changes in specificity [85]. Once that link is no longer the weakest, selection pressure shifts to another point in that physiological pathway such as the proteins of the CCM.

For a particular RuBisCO specificity, a CCM may be responsible for some of the variance in the K_o_/K_c_ due to its ability to boost the internal CO_2_/O_2_ ratio and maintain photosynthetic rates in the face of rising environmental O_2_/CO_2_. Although a CCM relaxes pressure for RuBisCO to have a high affinity for CO_2_ so K_c_ of C_4_ plants is generally higher than the K_c_ of C_3_ plants, but curiously there is no difference to the specificity of the RuBisCOs between the C_3_ and C_4_ plants despite the presence of a CCM. The higher K_c_ of the C_4_ plants is accompanied by a higher K_o_ presumably due to a relaxation in the binding for both substrates at the active site. Further, a more relaxed active site for each substrate, turns over those substrates faster so C_4_ plants require less nitrogen to achieve a given CO_2_ fixation capacity. But due to these correlative biochemical constraints, a CCM appears to yield no change in specificity over relatively short timescales.

In comparison, when considering the largely CCM-lacking Pavlovophyceae with the CCM-bearing Prymnesiophyceae of the haptophyte lineage, it is evident that the evolution of a CCM in the latter has both altered the substrate affinities and relaxed the specificity of the RuBisCO enzyme (Fig. 3). A possible explanation for the difference between the evolution of specificity in RuBisCOs of the haptophyte lineage and the C_3_/C_4_ plants is that it takes a long time to accumulate sufficient mutations to effect a change in RuBisCO specificity, on the order of 100s of millions of years. The signal of positive selection in haptophyte RuBisCOs at the branch between the Pavlovophyceae and Prymnesiophyceae [48] is dated to between 300 and 400 Ma. The step change in 1B RuBisCO specificity accompanies the divergence between the filamentous green algae and the land plants around 410 Ma, so about 200 Myrs after the rise to dominance of the chlorophytes ∼650 Ma [3]. By contrast, C_4_ plants have only been present for the last ∼30 Myrs [86]. It seems to take >100 Myrs to accumulate sufficient mutations to evolve an improved functional RuBisCO specificity.

These step changes in the RuBisCO specificity align with analyses of gene sequence for the RbcL gene across the algal phylogenies that find evidence for positive selection in the gene sequence clustered only at the base of the divergence of the modern algal lineages [48]. So RuBisCO undergoes major changes at the establishment of the haptophyte, and heterokont groups relative to the red algae [48]. This is consistent with the former use of RbcL genes for phylogenetic reconstruction i.e. as a species specific marker.

### 7.2 Fast evolution of Ko/Kc

By contrast, manipulations of the environment or intracellular O_2_/CO_2_ appear to yield changes to the RuBisCO substrate affinities at rates as fast as the timescale of decades. Assuming that the relative substrate affinity for RuBisCOs compensate for environmental O_2_/CO_2_ as proposed in Ref. [33], this timescale for adaptation derives from the degree to which these affinities appear to have kept pace with the documented changes in the environment. The K_o_/K_c_ of most C_3_ and C_4_ plants cluster at a level that could compensate for a Pleistocene glacial period when there was an excess of O_2_/CO_2_ between 39 and 50 fold (Fig. 2), or somewhere in between the extrema of the glacial-interglacial variance. It is reasonable to compare plant RuBisCO kinetics to dissolved gas ratios since RuBisCO experiences those substrates in a dissolved state. The K_o_/K_c_ of algal RuBisCOs appear better tuned to current conditions (O_2_/CO_2_ ratio of 13–19), a dissolved ratio that is lower than that experienced during the last 1 million years of the Pleistocene glacial cycles because of the anthropogenic rise in pCO_2_ over the last two centuries. Consequently the fine-tuning of RuBisCO's relative affinity for substrates appears to evolve in response to environmental change over timescales of tens of kyrs in the plants, and hundreds of years in the algae. This observation supports the emerging view that RuBisCO may be optimized to its environment [74]. Its kinetic performance can even be classified as moderately efficient when compared to a global overview of enzyme kinetic rates [[87], [88], [89]].

Adaptation processes may work differently in relatively small, subdivided populations of terrestrial organisms and astronomically large populations of marine phytoplankton inhabiting a fairly homogenous environment. Population size is one of the most important parameters that determines the amount of new genetic variation introduced into a population via mutation (the more individuals, the more copies of a gene in a population to mutate every generation), as well as the dynamics of spread and loss of the mutations by chance or selection [90]. It has further been shown that molecular evolution is proportional to generation time in plant lineages and microbial lineages [91,92]. Perennials, with longer generation times, have been shown to accumulate substitutions more slowly than rapidly maturing annual plants. It is likely therefore that the substrate affinity of algal RuBisCOs can be fine tuned to environmental change more quickly and potentially keep pace with anthropogenically diminishing O_2_/CO_2_ ratios, compared to the slower evolving plant RuBisCOs which appear to be stuck in glacial times. The rate of evolution of algal RuBisCOs is potentially orders of magnitude faster than that of plants due to their small generation time compared to that of plants. There is a hint that some diatom species (two strains of *P. tricornutum* and *C. fusiformis*) are also better adapted to glacial O_2_/CO_2_, potentially due to longer generation times as a result of resting spore formation, akin to the slower evolution rates in spore-forming bacteria.

## 8 Implications for optimizing photosynthesis

Photosynthesisers have evolved two strategies for achieving similar rates of net carboxylation at a given environmental O_2_/CO_2_. The evolution of a CCM maintains a more ancient O_2_/CO_2_ ratio, shielding the RuBisCO against the environment and keeping a lower specificity RuBisCO competitive for carboxylation (e.g. the cyanobacteria whom likely increase internal CO_2_ 10-fold above the environment) but at an additional nutrient cost. By contrast, the CCM lacking species succumb to the selection pressure of the environmental O_2_/CO_2_ resulting in a more specific RuBisCO (e.g. the coastal *P. lutheri*).

Given sufficient time (100s of millions of years), the persistent expression of a CCM reduces the specificity of RuBisCO so a natural limit emerges as to how far net carboxylation, and RuBisCO can improve, within the bounds of evolution. The brown algae, which diversified between 150 and 200 Ma, live ecologically at greater elevation relative to the tide than the red algae. They are distinct for employing iodinated peroxidases [93] which suggests that their more recent divergence has allowed them to take advantage of the rise in ocean iodate documented by the carbonates [7] as part of their antioxidant strategy, and they are well adapted to high O_2_/CO_2_. The brown algae could harbor the Rolls Royce of RuBisCOs, currently limited to this supratidal zone of hyperoxic conditions and are a worthy candidate of characterization of RuBisCO kinetics in the quest to find the most efficient RuBisCO.

At the other end of the spectrum, the cyanobacteria and green algae appear to have a RuBisCO which, as a bare enzyme, is poorly optimized for the modern oxygenated environment. Within the β-cyanobacteria, an exceptional CCM has evolved including the carboxysome (e.g. Ref. [94]) that compensates for the low specificity RuBisCO. But given that better RuBisCOs exist, has the improvement of RuBisCO within the cyanobacteria been limited by some other factor? It has been speculated that a first Calvin cycle might have evolved from ancient nucleotide metabolism and initially served in redox cofactor balancing and/or mixotrophy, before developing autotrophic function [11]. The 1B form of RuBisCO has also been invoked to be involved in anaerobic methionine sulphur salvage metabolism with the suggestion that the active site of RuBisCO has evolved to insure that this enzyme maintains both key functions [95]. Each of these RuBisCO functions may be lost with an evolution to a higher specificity RuBisCO. As a result the green algal and cyanobacteria RuBisCO may be limited to lower intracellular redox conditions that allows the maintenance of some anaerobic pathways to enable success in environments of fluctuating oxygenation.

## 9 Geological implications

A prolonged increase in dissolved ocean or intracellular O_2_/CO_2,_ can precipitate rapid change in the RuBisCO enzyme. Although it is tempting to speculate that improvements in RuBisCO might increase carbon fixation rates and oxygen production rates and set the atmospheric composition [96], the majority of change in RuBisCO is an adaptation to an environment which is less favourable to net carboxylation. Any adaptation in terms of specificity and/or induction of a CCM helps sustain carboxylation rates as the environment O_2_/CO_2_ becomes less favourable.

In terms of the ocean atmosphere budget of CO_2_ and O_2_, it is likely that the ratio underwent distinct step changes through geological history. The two atmospheric gases are inversely linked via the burial of carbon in its reduced organic carbon form, the dominant geological driver, that acts to decrease CO_2_ at the same time as driving O_2_ increase. An interrogation of a recent compilation of Phanerozoic δ^13^C of the ocean points towards a first order monotonic rise in ocean δ^13^C through the Palaeozoic indicative of an increasing proportional burial of organic carbon relative to carbonate peaking with the heaviest δ^13^C of the Phanerozoic ocean between 250 and 200 Ma [80,81]. The increase throughout the Paleozoic towards the Mesozoic parallels the tectonically paced aggregation of the Pangaean supercontinent and its migration away from the poles towards the low latitudes. It is likely then that either a large area of low latitude continental shelf buried a significant proportion of reduced carbon, or the amalgamation of plates uplifted significant shelf organic carbon onto the continents and out of the geological carbon cycle. Alternatively, submarine fans that accumulate during mountain erosion are efficient at burying large quantities of organic carbon from a vegetated land surface [97]. Pangaea was the first time in geological history that the continents amalgamated, contained significant mountains after plate collision and were covered by terrestrial biota. This maximal sink of organic carbon could have shifted the redox balance of the atmosphere/ocean towards a final rise in atmospheric O_2_ and lowered CO_2_. It may have been this peak organic carbon burial that tipped the environmental balance in the surface ocean towards the chlorophyll *c* containing lineages and propagated the positive feedbacks towards deepening OMZs, increasing pH and enhanced oligotrophy. There is a hint that the first sedimentary evidence for the coccolithophores (∼220 Ma), and the biomarker change may have predated the deepening of the OMZ by ∼ 20 million years (Fig. 1 [98]).

### 9.1 The deepening of the OMZs

A small trigger such as those described above can easily propagate to a large selective force by a positive feedback and co-evolution between the environment and phytoplankton physiology at the start of the Mesozoic. An increase in the redox or Eh of the upper ocean, or a deepening of an OMZ goes hand in hand with an increase in pH of the surface ocean [99] due to the deepening of the oxidative remineralisation of organic matter. So this persistent oxygenation of the upper waters also accompanied a persistent alkalinisation of the surface ocean helping the advent of mineralised skeletons and carbonate buffering of the deep ocean [100]. There is a positive feedback between the deepening of the oxygen minimum zone due to the enhanced ballast [7,101], increased alkalinisation of the surface ocean, aiding calcification and contributing to the persistent oxygenation. Such ballasting also deepens nutrient remineralisation leaving the surface ocean increasingly depleted - a condition less tolerated by the more nutrient hungry cyanobacteria and green algae that flourish under eutrophication than the nutrient-lean red algae. Concurrently the macro fauna themselves may not just be recipients of additional energy, but by their change in lifestyle as a result of the increasing transfer of nutrients from the lower echelons of the ecosystem they may also be implicit in driving the deepening of the OMZs and oxygenation of the upper ocean by their daily vertical migration [102]. There is a feedback loop between deepening OMZs, persistent oxygenation, alkalinisation, and oligotrophy which once set in motion creates an aggravating selective force towards the mineralising coccolithophores and diatom success over the incumbent green algae setting the scene for the advent of the modern ocean and its biota.

## 10 Conclusions

The different photosynthetic lineages, and their expressed RuBisCO specificities appear to evolve from contrasting redox endmembers towards similar “redox poise” under modern oxygenated conditions. The view of RuBisCO kinetic data, here, and the fossil record suggests that the red algae and other lineages with red algal-derived plastids (e.g. haptophytes, heterokonts) with a superior RuBisCO specificity were restricted to the oxic intertidal oasis through the Paleozoic. A step change in upper ocean oxygenation, enhanced oligotrophy, and elevated surface ocean pH at the start of the Mesozoic allowed them to inundate the open ocean. By contrast, the β-cyanobacteria and green algae with a greater nutrient requirement to support the CCM supply of carbon for their lesser specificity RuBisCO were restricted to the nutrient rich coastal ocean. This photosynthetic strategy is better adapted to fluctuating anoxic conditions which may have been a key to their successful invasion of the land through periodically inundated and anoxic soils. We explore the tempo of adaptation within RuBisCO kinetics to changing environmental O_2_/CO_2_ ratios and show that evolution of a CCM, at a nutrient cost, acts to relax RuBisCO efficiency and so imposes a natural limit to the improvement of RuBisCO over Earth's history.
